# Research on multi-dimensional reconstruction mechanism of cloud native full link in the metaverse scenario

**DOI:** 10.1038/s41598-023-48724-y

**Published:** 2023-12-12

**Authors:** Shuo Sheng

**Affiliations:** Key Laboratory of Embedded System and Service Computing, Ministry of Education, Tongji University, Shanghai, China

**Keywords:** Astronomy and planetary science, Energy science and technology, Engineering, Materials science, Mathematics and computing, Nanoscience and technology, Optics and photonics

## Abstract

Recently, the Microservices whole link whole life cycle optimization framework has been a research hotspot in the academic and engineering fields, especially how to encapsulate the component security mechanism and conduct high concurrency testing based on the existing active framework. The whole link delay, load, and cost have always been the key optimization goals of Microservices splitting and deployment logic. Based on this, this paper proposes a multi-objective optimization Microservices framework that takes into account the security mechanism, Define the fitness function, define the upper and lower limits, and perform multi-dimensional constraints to filter for global and local optima. At the same time, reconstruct the circuit breaker current limiting mechanism, dynamically detect protocol parameters, and perceive the logical relationship of heartbeat status in real-time. Experimental results have shown that this innovative framework can solve scenarios such as high concurrency, high reliability, and high availability Performance bottleneck, business degradation, especially in financial and securities scenarios.

## Introduction

With the popularity of Microservices, every Internet company has numerous services of all sizes in the background, and there are countless invocation relationships between services. To ensure the maturity and stability of the entire Microservices system, it is necessary to ensure the maturity of each Microservices. But how to define the maturity of a service? What latitude should we consider? What are the common problems in various latitudes? How to optimize?

With the development of mobile Internet and Internet plus, the original SOA architecture has encountered four problems: ① lack of effective service governance, mixed service assets, and no effective service management and control means; ② The business support response is slow, and the system is too big to fail, unable to achieve real-time updates and modular release; ③ Poor system availability, unable to achieve 7 × 24 h uninterrupted service; ④ Innovative business is difficult to support, especially innovative business with Internet characteristics. Based on this, developers propose an SOA architecture based on Microservices with the starting point of architecture optimization^1–6^; Its core concept is to decompose complex application systems into multiple services in the form of independent business units, each of which can adopt different implementation technologies and be independently designed, developed, and deployed in a lightweight and more flexible mode, running in independent processes to form highly cohesive autonomous units.

Specifically, SOA divides the system into different services, uses interfaces for data interaction, and services are interdependent and effectively integrated to achieve the overall function of the system^7,8^. In terms of architecture, the Microservices architecture is an improvement based on SOA architecture, and incorporates the idea of componentization and domain modeling. First, the system is divided into multiple Microservices, which are deployed independently in a Loose coupling manner; Secondly, each Microservices only needs to complete its own tasks with high quality, and each task represents a fine-grained business capability; As a result, various businesses have been completely componentized and service-oriented^9–12^; Finally, modules that provide domain service capabilities realize service composition and assembly in the underlying Microservices architecture. As shown in Fig. 2a, the overall architecture puts all software functions in a process, and multiple servers jointly support the running of computing tasks, and finally returns the running results to the user; The Microservices architecture (Fig. 2b) decomposes the overall function of the software into multiple services supported by different types of servers; Then, the data is fed back to the database, and users can obtain data from the database, which not only speeds up the overall response speed of the system, but also meets the business needs of front and back end separation under the Internet environment. It can be seen that the advantages of the Microservices architecture^10^ are mainly reflected in: distributed (physical deployment, service deployment, data storage), high availability (distributed architecture, clustered deployment, automatic service registration) Scalable (on-demand resource allocation), intelligent operation and maintenance, and other aspects.

With the increasing expansion and complexity of software system functions, the SOA architecture based on Microservices has begun to rise. Specifically, Microservices supports the rapid response and personalization of business in the foreground. It uses service-oriented development SOD (service-oriented development), which is characterized by rapid development, timely response, easy implementation, etc.^13^; The ESB service bus, as the back-end, supports the integration of various systems, technologies and platforms, while the service-oriented infrastructure (SOI)^14–16^ has the characteristics of stability and high integration. The Microservices technology and ESB technology complement each other and play different core roles in the SOA architecture to achieve the self-service effect of software systems.

This article introduces a model used by the technical product teams of mainstream frontline large factories in the financial field to measure service maturity, and evaluates multiple backend services based on this model. It summarizes some common low sub items and organizes relevant optimization solutions for the low sub items.

## Optimization model

### Service maturity model

**Figure 1:**
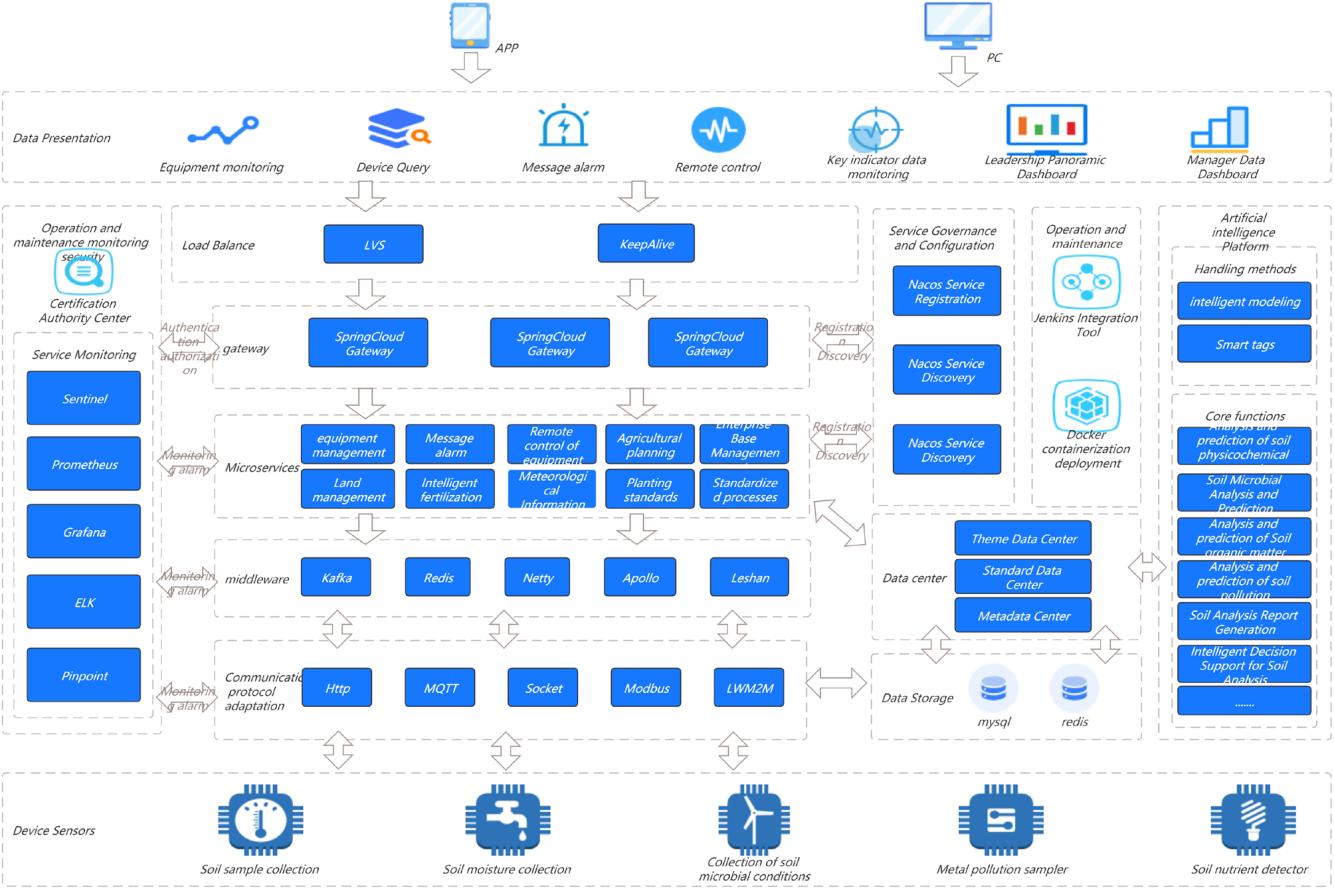
Full-link microservice security link topology.

In Fig. 1, in order to quantify the maturity of services, this study needs to evaluate services from multiple dimensions. Each service iterates continuously with the requirements, and each iteration cycle needs to go through the following three stages: development testing, operation and maintenance online, and online operation. At each stage, a series of measures are needed to ensure the final maturity and reliability of the service. This study focuses on each stage and identifies the indicators that need to be addressed to measure the overall quality of that stage. This study comprehensively rated the above three stages and graded service maturity based on the ratings. (1–5 points are classified as admission level, 6–7 points are classified as service level, and 8–10 points are classified as maturity level.) Through such a service maturity model, this study can intuitively evaluate the maturity of a service using quantitative indicators and identify areas for improvement.

### Low item sorting

Based on the service maturity model, this study evaluated multiple backend services and found that some indicator items generally scored lower in each service. These indicators include: development testing: code specification, unit testing, stress testing, interface testing; In terms of operation and maintenance online: grayscale online, online rollback; In terms of online availability: system protection, data backup, alarm processing, and emergency plans. Therefore, this study prioritized the optimization schemes for these low sub items, hoping to improve the maturity of services and elevate the services of this study to a mature level through the optimization of low sub items.

### Low sub item optimization plan

**Figure 2:**
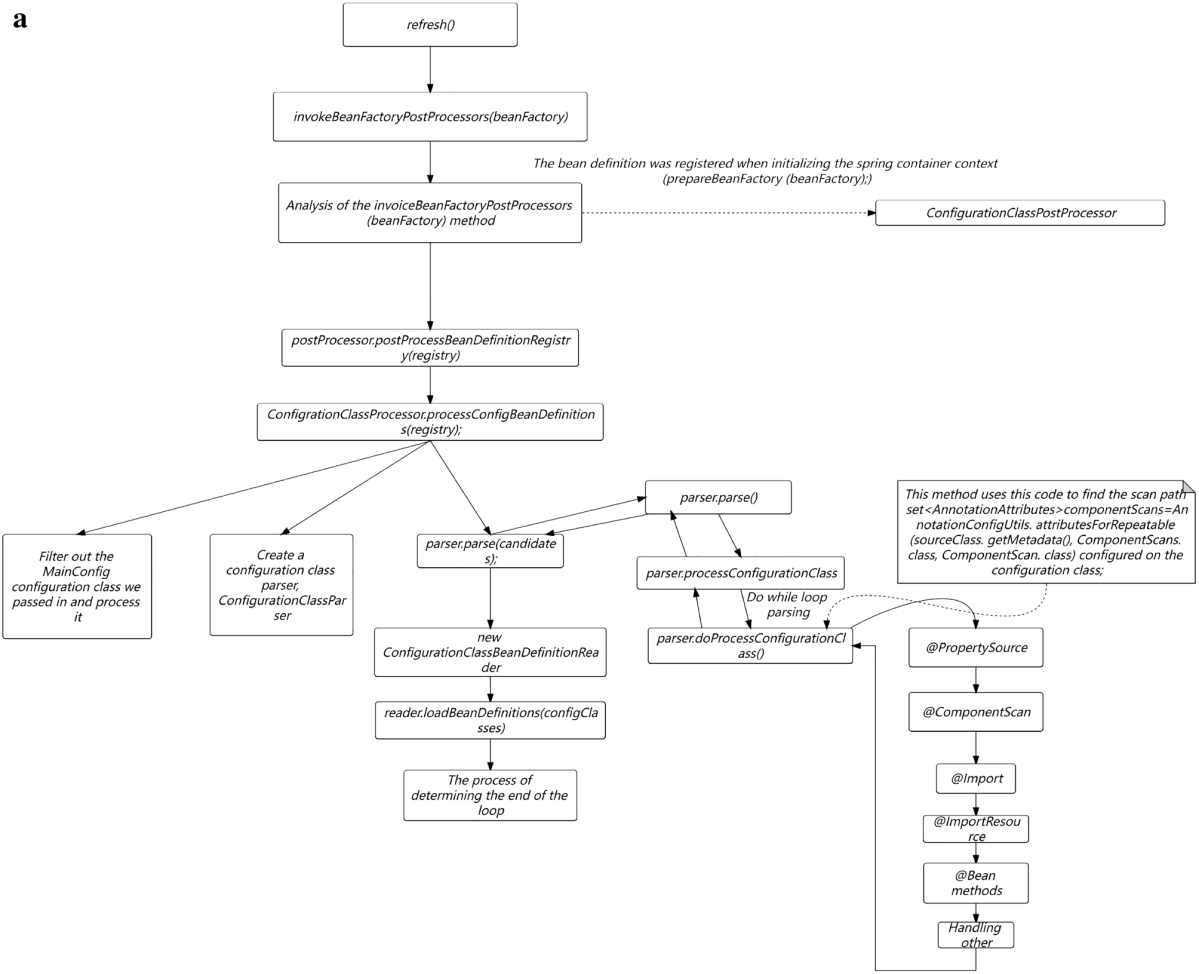

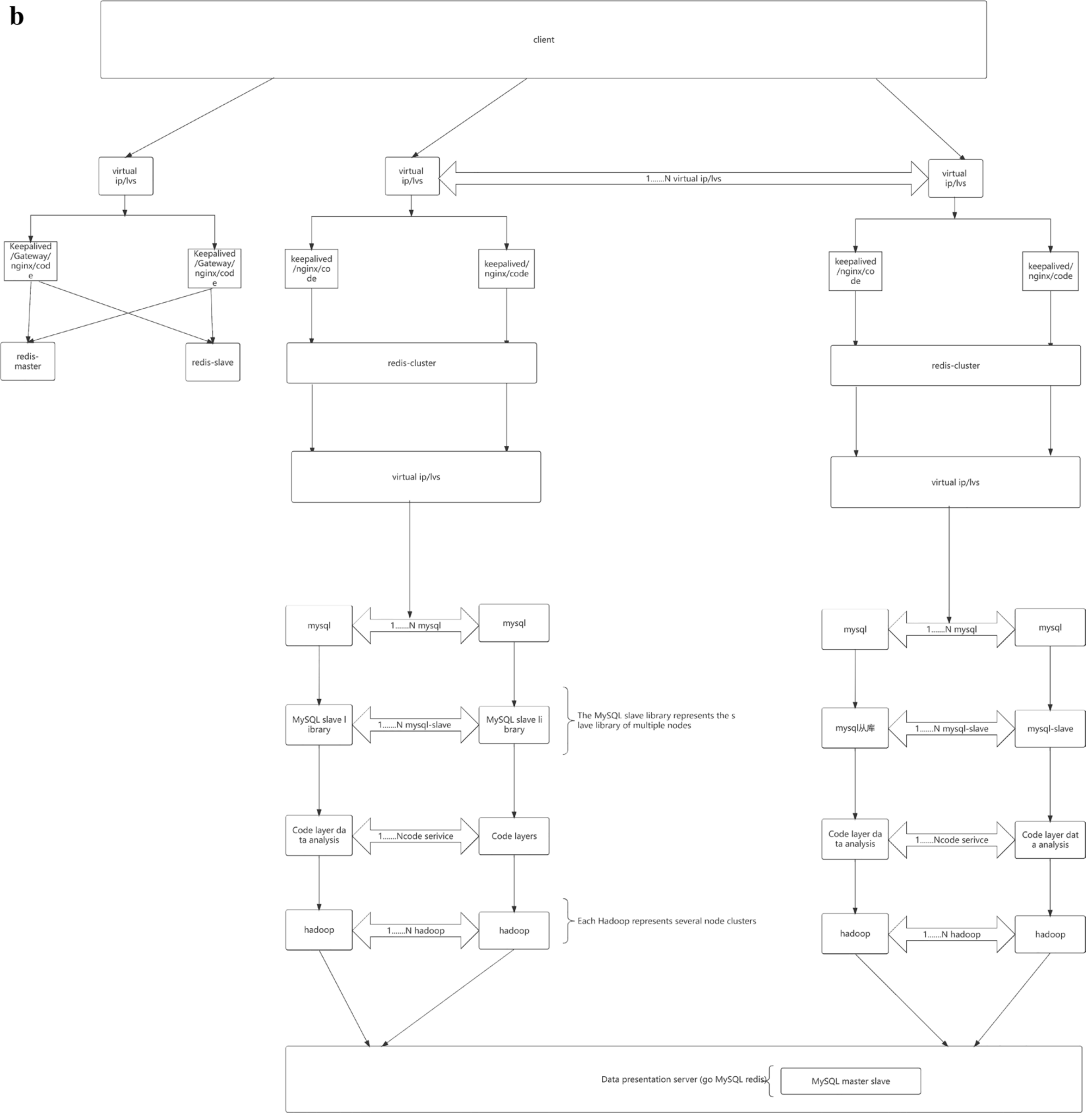
(**a**) Full link testing core routing. (**b**) Full Link Metaverse Cloud Native Data Business Logic Flow View.

In Fig. 2a,b, The services in this study are mainly developed based on the Java language, mainly using virtual machine deployment and a small amount of container deployment. So the optimization plan below mainly revolves around the current service status of this study.

### Development testing

Online bugs are often introduced by developing new requirements. If problems are discovered as much as possible during the development and testing phase, improving code quality can minimize the impact of the problem. Here are some common measures and means during the development and testing phase:

#### Code specification

Programming style. Collaborating with GitLab CI and Sonar inspections, it is possible to conduct code specification checks on each submission and present them visually in the form of reports.

#### Unit testing

In Fig. 2a,b, Unit testing is located at the bottom of the testing pyramid. Fast running efficiency, typically completed test cases run for no more than 10 min, and can be integrated into the CI process to quickly provide feedback on issues. Low maintenance costs, unit testing can detect problems during the development phase, and repair costs are low. The longer the feedback time for testing the pyramid, the higher the cost of implementation. Therefore, it is recommended to increase the coverage of unit testing.

The most commonly used testing framework for Java is Junit. In addition, in conjunction with the Mock testing library Mockito and the testing coverage tool Jacobo, a complete set of unit testing tools is formed. Jacco plugins can be configured in both IDEA and SonarQube to visually display the coverage of unit testing.

In the process of promoting unit testing, the common problems encountered in this study are: supplementing unit testing for projects is time-consuming and laborious, and how to control the testing coverage of new code?

**Figure 3:**
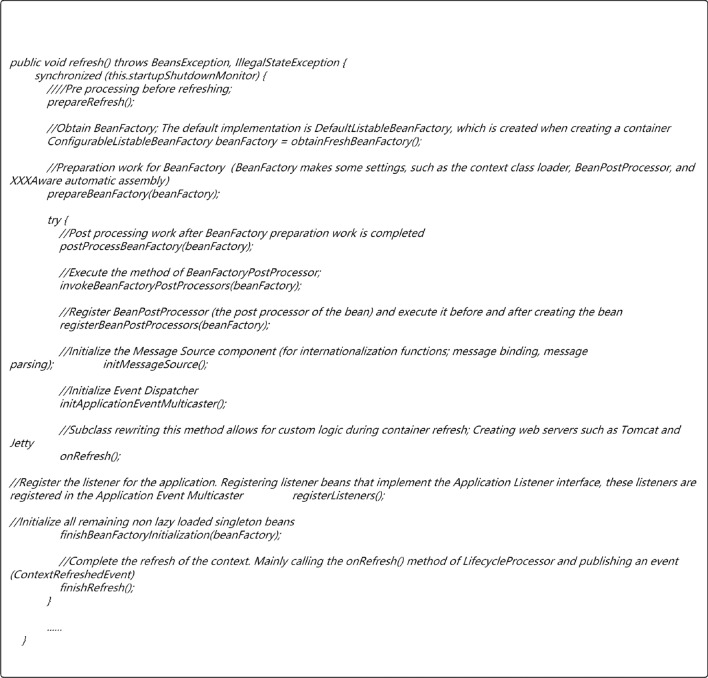
Optimization of core branch business timing.

In Fig. 3, the solution of this study is to prioritize supplementing unit testing for important code (such as public libraries and tool classes) with existing code. Indicators for increasing Code coverage of new code (e.g., set the test coverage of new code to be no less than 90%); As new code is added and old code is modified, the overall testing coverage of the project will gradually increase. Add code testing coverage statistics: Using the diff cover tool, you can calculate the testing coverage of the current branch's modified code by analyzing branch code differences and Jacob test reports.

#### Unit testing

1. Why do I need to do stress testing Through stress testing, this study was able to: identify performance bottlenecks; Estimate resource usage; Provide reference for current limiting configuration; Identify issues introduced by new requirements.
2. What functions should stress testing tools have Common stress testing tools include JMeter and others. This study uses the LoadMaker cloud pressure testing system provided by mainstream cloud platforms in the financial field of first tier large enterprises, which has the following characteristics: multiple pressure clusters, and the pressure can reach high levels; Create and save pressure testing scenarios, repeat execution; Provide pressure test reports: QPS, response time, error rate, and other data; Meanwhile, in order to ensure that pressure testing does not affect the actual traffic of online users, this study constructed a separate pressure testing resource pool. When pressure testing is required, selecting machines and pressure testing modules in the resource pool can automatically build a pressure testing environment.
3. When is pressure testing required? Routine pressure testing: Conduct routine pressure testing on key projects during the grayscale period of the version; Pre launch pressure testing for new projects/interfaces: Pre launch pressure testing for new projects to guide capacity estimation and current limiting configuration.

#### Interface dial test

**Figure 4:**
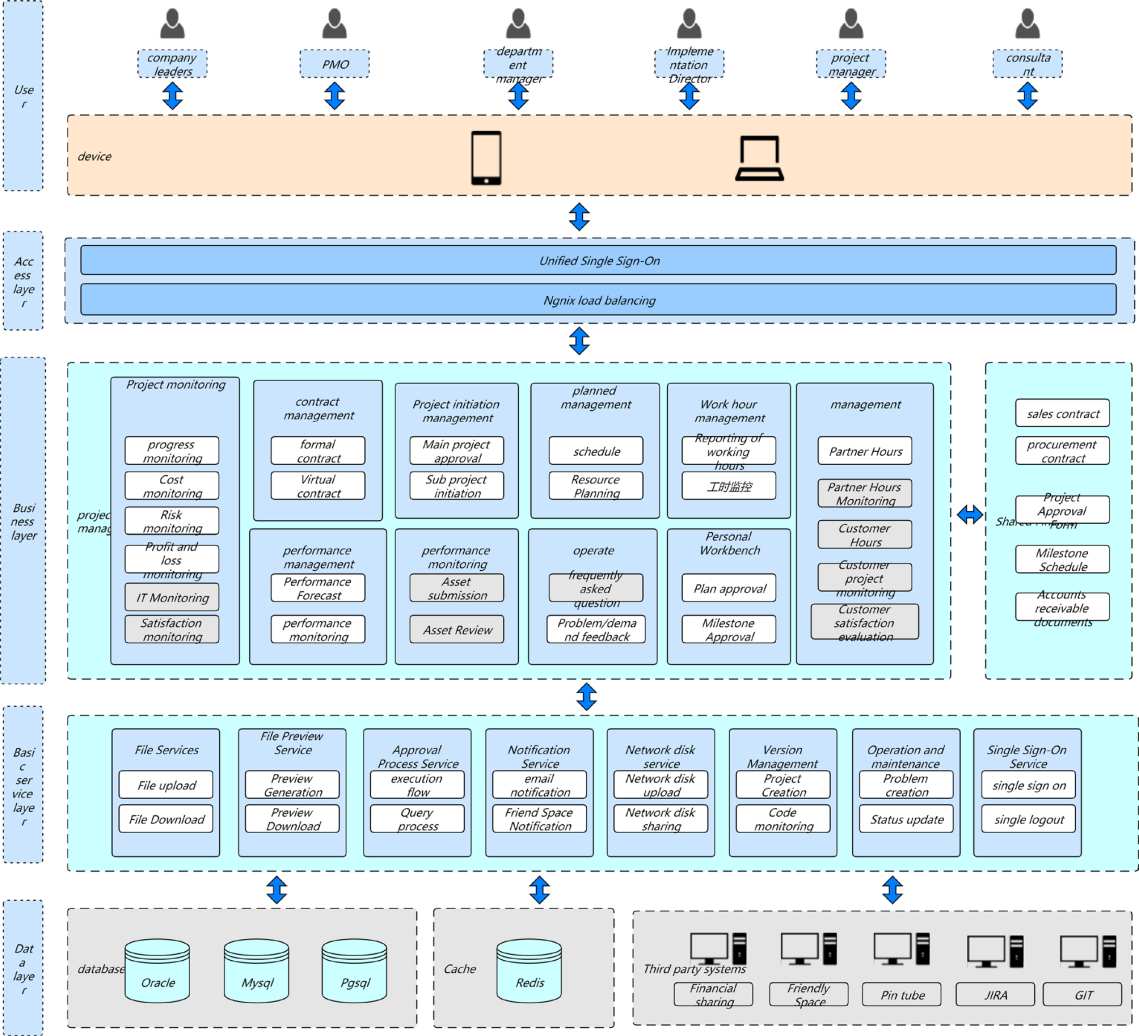
Full link security performance testing.

In Fig. 4, for backend interface services, this study is more concerned about whether the data returned by the online interface is correct and whether the new code has affected the existing logic? Interface testing can effectively cover the above two issues. On the one hand, it can be used for verifying the data returned by online interfaces and detecting interface anomalies in real-time. On the other hand, it can be used to automate the API testing, cooperate with the online process, find out whether the new code is introduced, and terminate the online operation in a timely manner. An interface testing system should have the following features: support verifying the interface return results; Complex verification supports writing scripts; Multiple test points can be set to simulate formal user request scenarios; Support for alarms; Detailed test reports; Get through with the online process, and support automated API testing after grayscale.

### Operation and maintenance launch

#### Grayscale online mechanism

Passing code testing in a testing environment does not guarantee that the code has no problems at all, and there is still a possibility of undetected problems caused by testing omissions or environmental differences. If there is no grayscale online mechanism, these potential issues can directly go online, which may have catastrophic consequences for the online system. The grayscale launch needs to be accompanied by grayscale checks to ensure the safety of this launch. The inspection methods used in this study include: automatic interface detection for grayscale machines after grayscale is brought online; Compare the monitoring indicators of grayscale machines with those of online machines; Acceptance of grayscale environment testing function.

#### Online rollback

If an online problem occurs due to the launch of new code, the first thing to do should be a rollback operation, rather than analyzing the problem and fixing the code launch. The rollback operation needs to achieve the following points as much as possible: the rollback speed is fast, and if it is necessary to reverse the code, recompile and package it, and upload it to the server, the entire process will be relatively long, and the fault recovery time will also be relatively long; Rollback is complete, and some code and configuration files are dependent. If only the code is rolled back, it may cause exceptions after startup; Rollback capability requires architectural consideration to avoid situations where rollback is not possible, such as data writing after going live, which is incompatible with the rolled back code.

This study adopts the RPM packaging method for online and rollback. Package all the content (code and configuration) deployed at once in the form of RPM. During deployment, the server retains the historical version of the RPM package. When rollback is required, simply execute the Job to install the historical version of the RPM package. If the service is deployed using a container, it will be easier to roll back by simply starting the mirror of the historical version.

### Online availability

#### System protection

**Figure 5:**
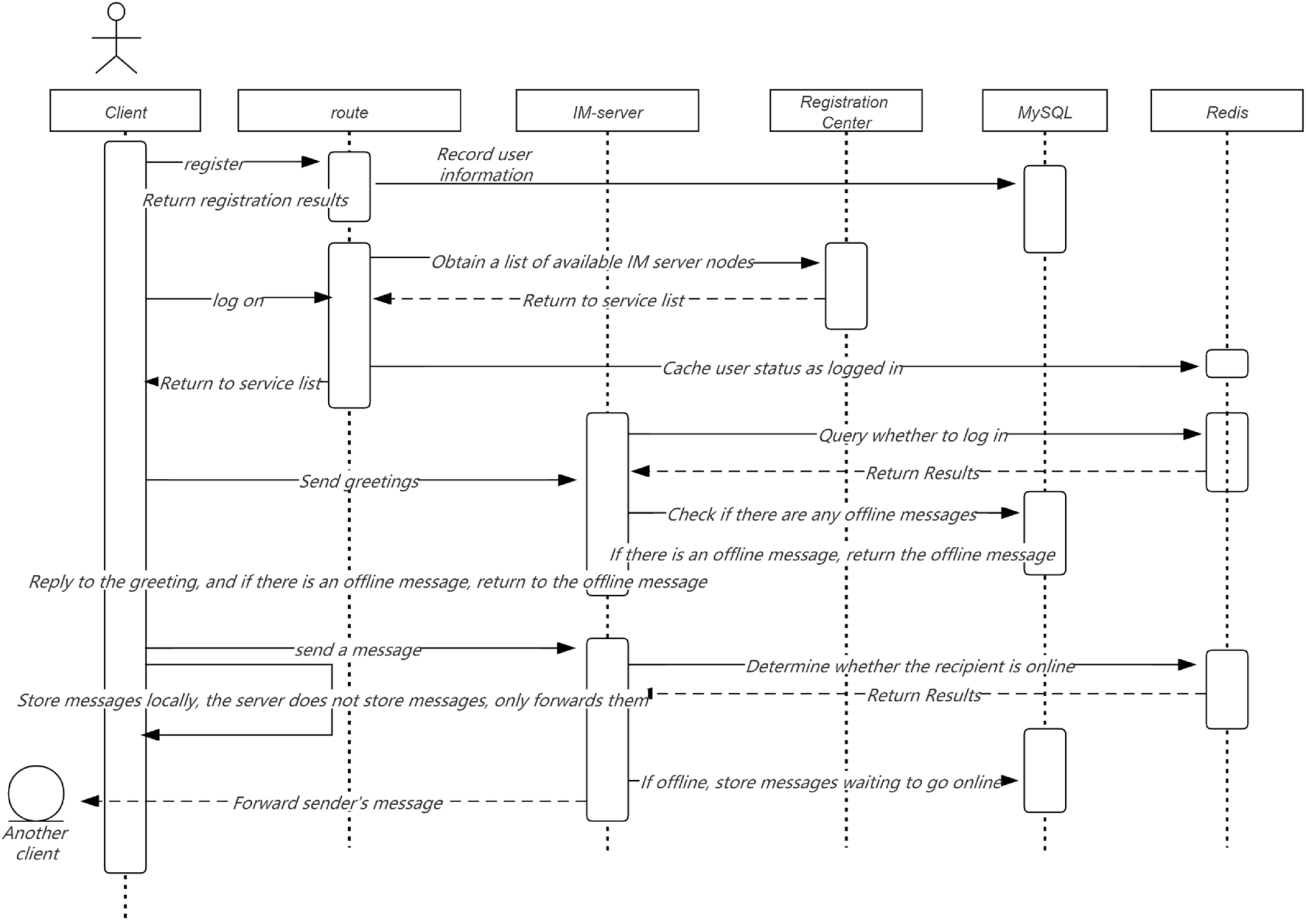
Full link security and effective timing.

In Fig. 5, online operation may face various problems, such as machine failures, network issues, dependent service provider crashes, and a surge in traffic. How to survive in such a dangerous environment? We cannot trust others, we can only improve the reliability of our services. The general methods adopted include: circuit breaker, current limiting, degradation, retry, etc.

Circuit breaker: When there is a problem with the service provider that this study relies on, the service of this study cannot be dragged down. The commonly used method is circuit breaker. When the success rate of the relying party is lower than the threshold set in this study, pause the call to it and retry after a certain interval. Commonly used fuse components include Hystrix, Sentinel, etc. The following table is a comparison between Hystrix and Sentinel. Considering that Netflix no longer maintains Hystrix and Sentinel has more diverse functions, this study chose Sentinel as the fuse component for this study^22–26^.

Downgrading: When a sudden accident occurs and the overall service is overloaded, in order to avoid overall unavailability of the service, service degradation is generally enabled to ensure the normal operation of important or basic services, and non important services are delayed or suspended. Therefore, this study implemented a page degradation service. The service will periodically request the page interface and verify it. After passing the verification, the results will be saved; When the service encounters abnormalities or overload, turn on the downgrade switch, and the page service will directly read the previously saved static page data and return it. Sacrifice personalization and downgrade from thousands of people and faces to thousands of people and faces. Due to the removal of the page construction process, the processing power will be greatly improved to ensure the normal operation of the service^27–31^.

**Figure 6:**
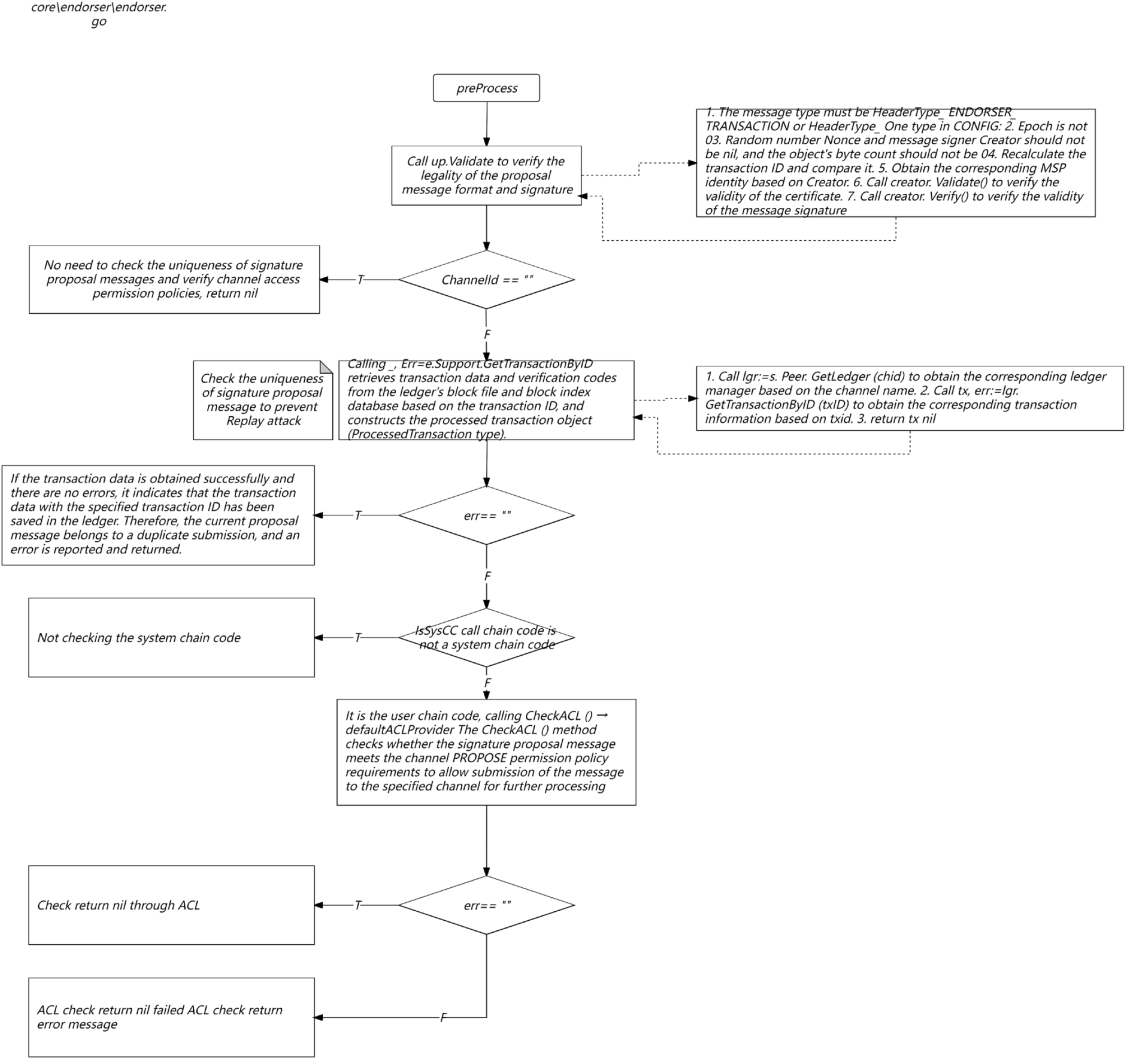
Full Link System Security Routing Optimization.

In Fig. 6, current limiting: Current limiting is very important as it is an important level for protecting the normal operation of services. Especially in the event of a sudden increase in traffic, reasonable flow limiting can protect your service from being overwhelmed. If flow restriction is not configured, not only will the service be disrupted during a sudden increase in traffic, but it will also be difficult to recover because the service will be disrupted again after restarting. This study generally configures current limiting at the gateway layer and service layer separately. The gateway layer can configure single machine current limiting using nginx's current limiting plugin. The service layer uses Sentinel flow limiting. Sentinel can limit traffic based on QPS, concurrency, and call relationships, and supports overall cluster traffic restriction. The following figure shows the implementation of Sentinel cluster current limiting.

**Figure 7:**
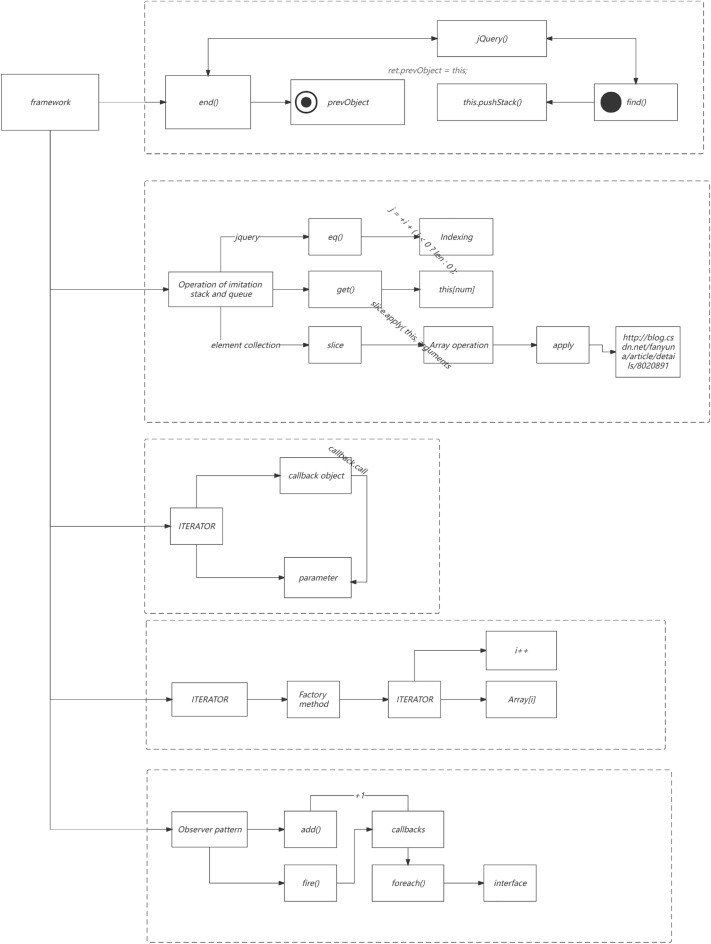
Core data security flow.

**Figure 8:**
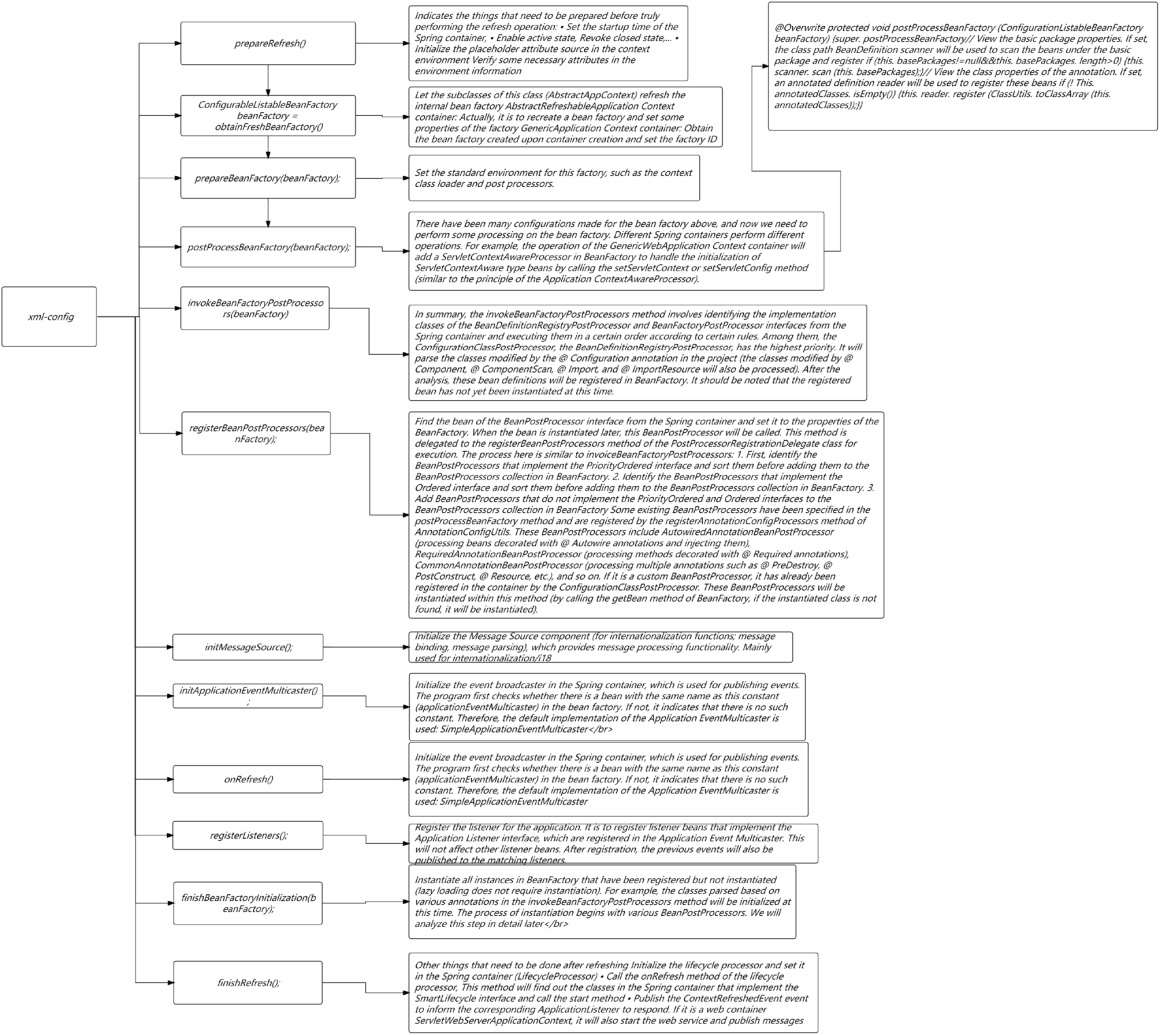
Time series flow native distribution mechanism.

In Fig. 7, Try again: From the client to the backend service, there may be exceptions in various stages, such as DNS failures, operator node failures, backend server failures, etc., resulting in request failures. For different situations, appropriate retries can increase the success rate of requests. There are several types of retries on the mainstream frontline financial industry APP end:

In Fig. 8, IP direct retry, which controls the number of retries by configuring the number of direct IPs; Super pipeline retry, the company's self-developed HTTP based gateway proxy service, which can achieve remote retry; HTTP retry; Try again with the original URL.

Retries can improve the success rate, but excessive retries may lead to excessive pressure on back-end services and Avalanche effect. So the following points should be noted in the retry strategy: the retry strategy can be cloud controlled, and in extreme cases, it can be configured in the background to turn off retry; Distinguish error codes and try again. Not all error codes need to be tried again, such as error codes returned by backend triggering current limiting, which do not need to be tried again.

#### Data backup

In terms of database usage, this study needs to consider the high availability of the database. In this regard, the company's service cloud team has made many optimizations, which can be found in this article. This article will not be elaborated on. As a business unit, it is important to note that the use of databases should strictly follow the architecture of two locations and three centers for application and use.

#### Monitoring alarms

A comprehensive monitoring and alarm system can enable this study to detect and handle service issues in a timely manner, avoiding their spread. From different latitudes, this study requires different monitoring, mainly including:

Indicator monitoring; The key indicators of monitoring services include three parts: machine indicators (CPU, memory, network traffic, etc.), service indicators (QPS, success rate, response time, etc.), and third-party interface indicators (QPS, success rate, response time, etc.).

The specific implementation plan is to report indicators through Collected and self-developed Meerkat components, submit them to Graphint for summary and storage, and display them through Grafana.

Application log monitoring: Based on application log monitoring, this study can discover more fine-grained exceptions, such as exceptions thrown within the application, internal business logic errors, etc. Firstly, this study defines a format specification for log printing, where all logs that need to be monitored are output in a unified format. Then, through the log collection component Venus, the logs on the machine are delivered to the Kafka queue, and finally entered into the Druid temporal database for multi-dimensional analysis and storage. The front-end report is presented using Graphna^17–20^.

Full link monitoring: If you want to monitor contextual link relationships, cross system fault localization, and other related issues, you need to perform full link monitoring. For detailed information, please refer to the article "Exploration and Practice of Full Link Automated Monitoring Platforms in the Financial Field of Mainstream First tier Large Factories" in this article.

#### Emergency plan

Although a series of availability optimization measures were listed above in this study, various anomalies and failures still occur in real online environments, requiring manual handling in this study. When a problem truly arises, if you go to the scene to think about how to handle it, you may not have achieved the optimal solution due to panic. Therefore, this study needs to anticipate potential issues and how to handle them in advance, and form a written document. When problems arise, this study only needs to process the document step by step^21^.

### Service quality inspection

With an optimization plan in place, how can I determine if the service has been implemented according to the optimization plan? How to ensure high long-term availability of services? This study does not want to complete a round of optimization. After the service maturity is improved, if there is no follow-up for a long time, the service maturity will gradually begin to decline.

Therefore, automated checks were conducted on these common service maturity indicators, and daily health reports were generated to monitor the maturity of services. Once there is an abnormality in a certain indicator, this study can handle it in a timely manner. This keeps the service maturity of this study in a long-term stable state.

## Prototype experiment

Modeling prototype:

$$ \begin{gathered} \min f_{1} = \sum\limits_{j = 1}^{n} {\omega_{j} } *T_{j} \hfill \\ \min f_{2} = 10*\log \frac{{\sum\nolimits_{i = 1}^{m} {\sum\nolimits_{j = 1}^{n} {\sum\nolimits_{p = 1}^{n} {\sum\nolimits_{\nu = 1}^{{u_{i} }} {10^{{0.1*PN_{i\nu } }} } *\frac{{l_{j} }}{{V_{i\nu } }}*x_{jipv} } } } }}{{\sum\nolimits_{i = 1}^{m} {\sum\nolimits_{j = 1}^{n} {\sum\nolimits_{p = 1}^{n} {\sum\nolimits_{\nu = 1}^{{{\text{u}}_{i} }} {\frac{{l_{j} }}{{V_{i\nu } }}} } *x_{jipv} } } }} \hfill \\ \min F(x) = [f_{1} (x),f_{2} (x),...,f_{m} (x)] \hfill \\ s.t.\left\{ \begin{gathered} g(x) \le 0,i = 1,2,...,p \hfill \\ h(x) = 0,j = 1,2,...,q \hfill \\ \end{gathered} \right. \hfill \\ \end{gathered} $$

**Figure 9:**
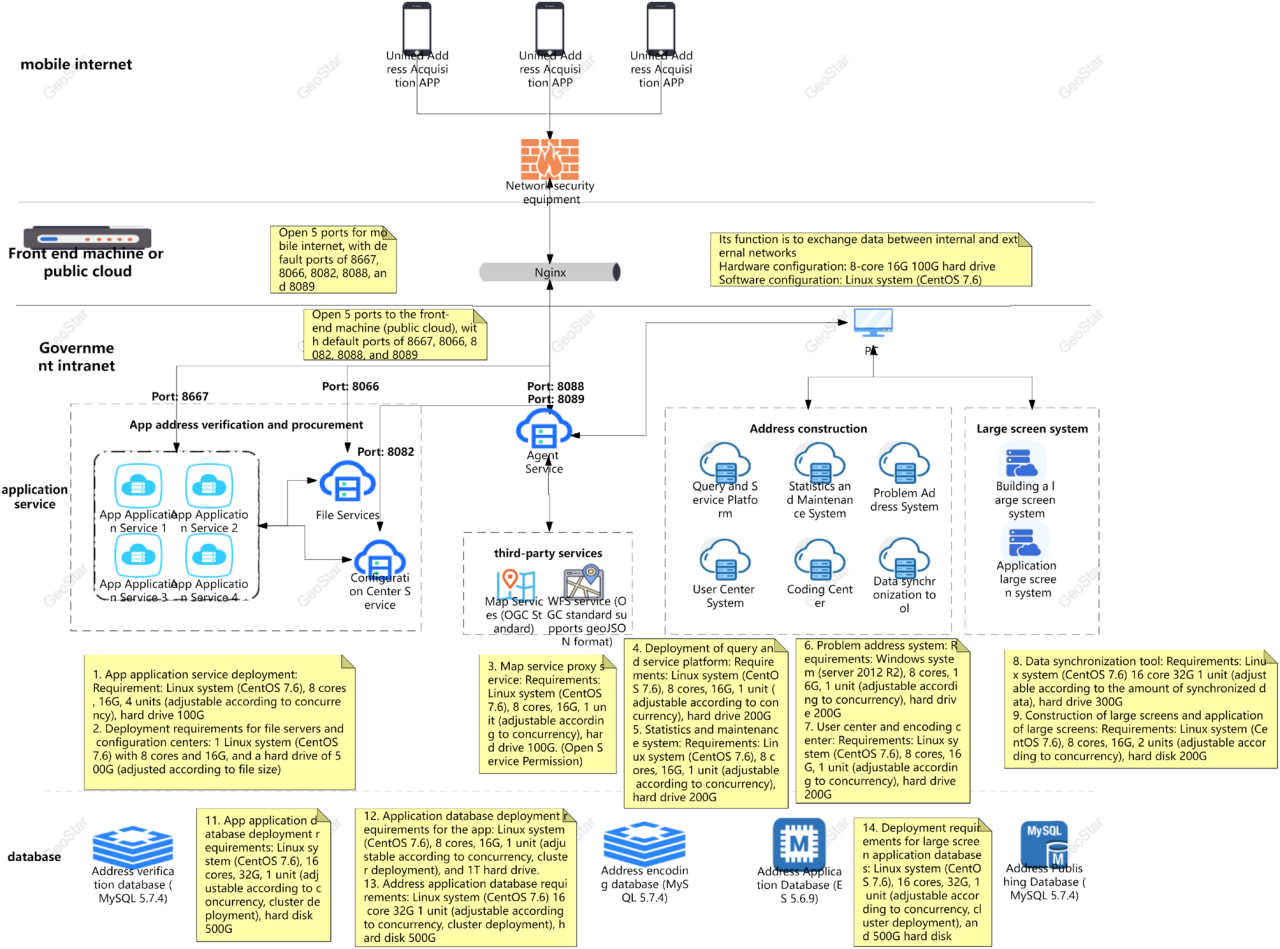
Full Link Technical Architecture for Production Environment.

As shown in Fig. 9, the native temporal and global architecture of the metaverse full link is shown in the figure. From the UE end to the firewall, the load balancing agent optimizes multi-dimensionally, accesses the port port and IP whitelist based on REST, and performs multi-dimensional DDD partitioning. At the same time, the upper and lower boundaries are clearly defined, including third-party services and configuration file collections. The architecture is deployed in the form of multiple nodes, multiple live in different places, and dual live in different places, The database adopts a hybrid approach of static and dynamic multidimensional filtering for persistence. The specific network protocol, data direction, logical architecture, and parameter configuration are shown in the figure. Containerization: To achieve environmental consistency and rapid deployment of cloud native architecture, containerization technology is used to package applications and their dependencies into independent containers, achieving environmental consistency and rapid deployment. Containerization enables applications to maintain consistency in different environments, and containers can be quickly deployed and started, accelerating application delivery speed.Metaverse Cloud Native Architecture: To achieve flexibility and scalability, the cloud native architecture adopts a microservice architecture that splits applications into small, autonomous microservices. The microservice architecture enables different parts of an application to be independently developed, deployed, and extended, achieving loose coupling and flexibility. Each microservice can independently scale according to demand, providing better ability to cope with high loads and peak traffic.

Continuous delivery and integration: Achieve rapid feedback and delivery. The cloud native architecture supports the practice of continuous delivery and integration, and achieves rapid feedback and delivery through automated construction, testing, and deployment processes. Continuous integration ensures that every code submission undergoes automated testing, improving software quality and stability. Continuous delivery achieves rapid delivery through automated processes, meeting the rapid changes in market demand and user feedback.

Automated management: Realize elastic scaling and fault recovery with cloud native architecture. Utilize automated management tools to achieve elastic scaling and fault recovery. Automated scaling automatically adjusts the number of application instances based on load changes, ensuring system resilience and high availability. Automated management tools can automatically configure and manage various components of an application, reducing manual operations and errors. At the same time, monitoring and automated fault detection tools can timely detect and handle application failures, and quickly recover.

**Figure 10:**
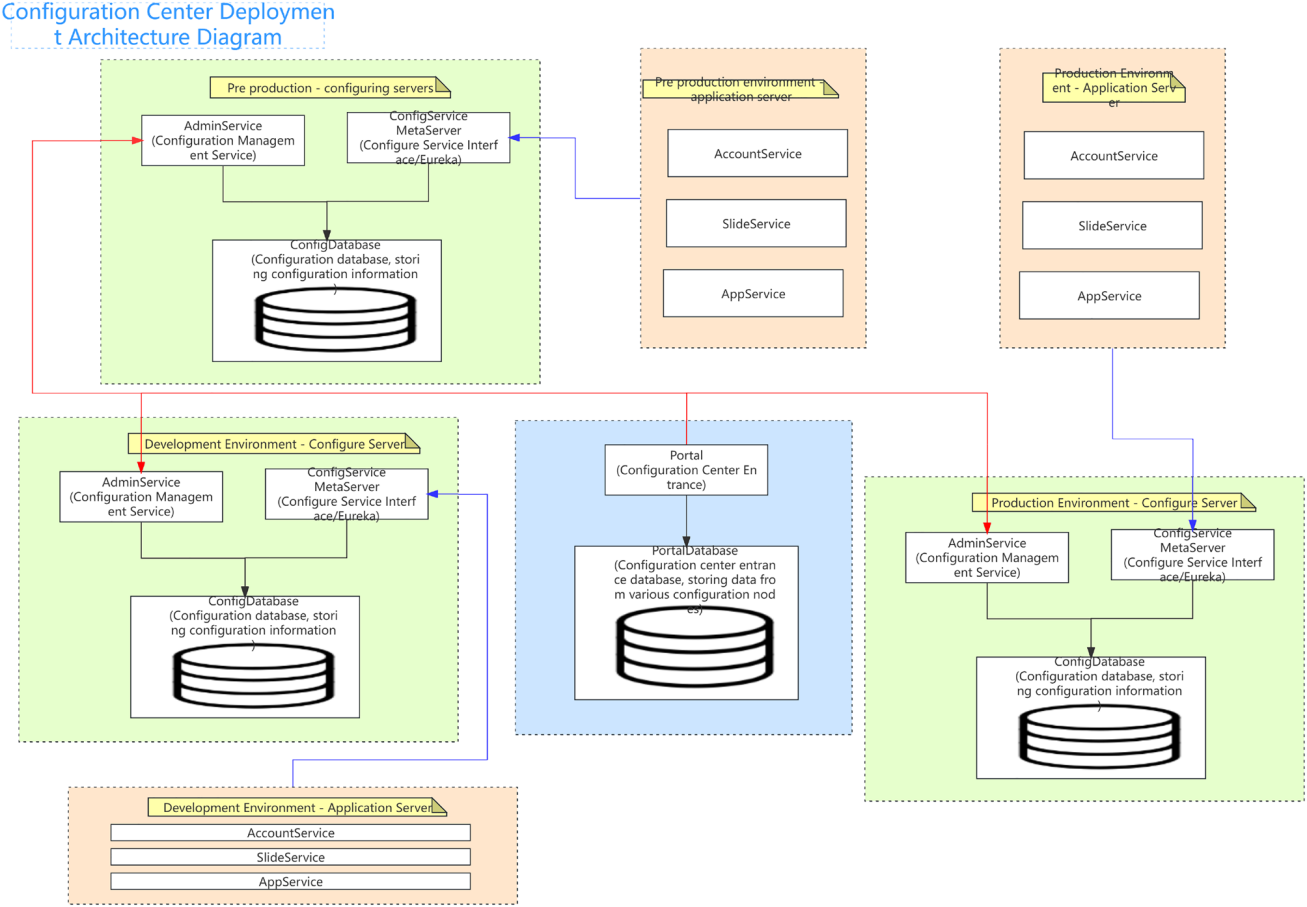
Full Link Data Storage Security Configuration.

As shown in Fig. 10, the database deployment architecture is shown in the figure, with hierarchical deployment persistence. In the configuration database, the binlog is divided into databases, tables, and mapped table fields. The parameters are dynamically transmitted to the application server and traffic model. The data link with the lower layer is read and written based on socket rest, and the DEV is not delivered in one click mode. The portal is responsible for attribute partitioning on the interface side and preprocessing of parameter proxies, The configuration server is responsible for encapsulating and distributing user role attributes and related business operations.

Distributed transactions are usually run on machines with multiple nodes, and there is a process of RPC during runtime. Compared to a single system database, there are more abnormal links in ensuring the atomicity of transactions, and there are more recovery and rollback situations. Currently, most of them are solved through two-stage submission protocols. When a distributed transaction is submitted to multiple servers for processing, each server needs to record logs. When a transaction fails, the corresponding server needs to perform a rollback operation, which is a typical distributed transaction processing process.

Upgrading from "two places and three centers" to "three places and five centers" is a major upgrade of infrastructure, not just an increase in simple data replicas. The architectural improvements it brings include the following points.

1. The database has urban level disaster recovery capabilities.
2. The application has urban level disaster tolerance capability, and the deployment of the application can achieve a dual center model of City 1 and City 2, enhancing the disaster tolerance capability of the application.
3. The capacity of the database has increased, the number of read-only replicas has increased, and service capabilities have been enhanced.

This upgrade also introduces new challenges to the original data center architecture.

1. The increased time consumption brought about by cross city has an impact on batch processing of business, overall link time consumption, and hot lines.
2. The number of data synchronization replicas has increased, and the network in the original machine room needs to be expanded.
3. The number of data synchronization replicas has increased, and the hardware resources of the original tenants have expanded.

During the process of architecture upgrade, it is necessary to always maintain disaster recovery capability: after any single computer room fails, the cluster remains available, and in case of a computer room failure in any city other than the city 1 where the main database is located, the cluster remains available and can still provide services.

**Figure 11:**
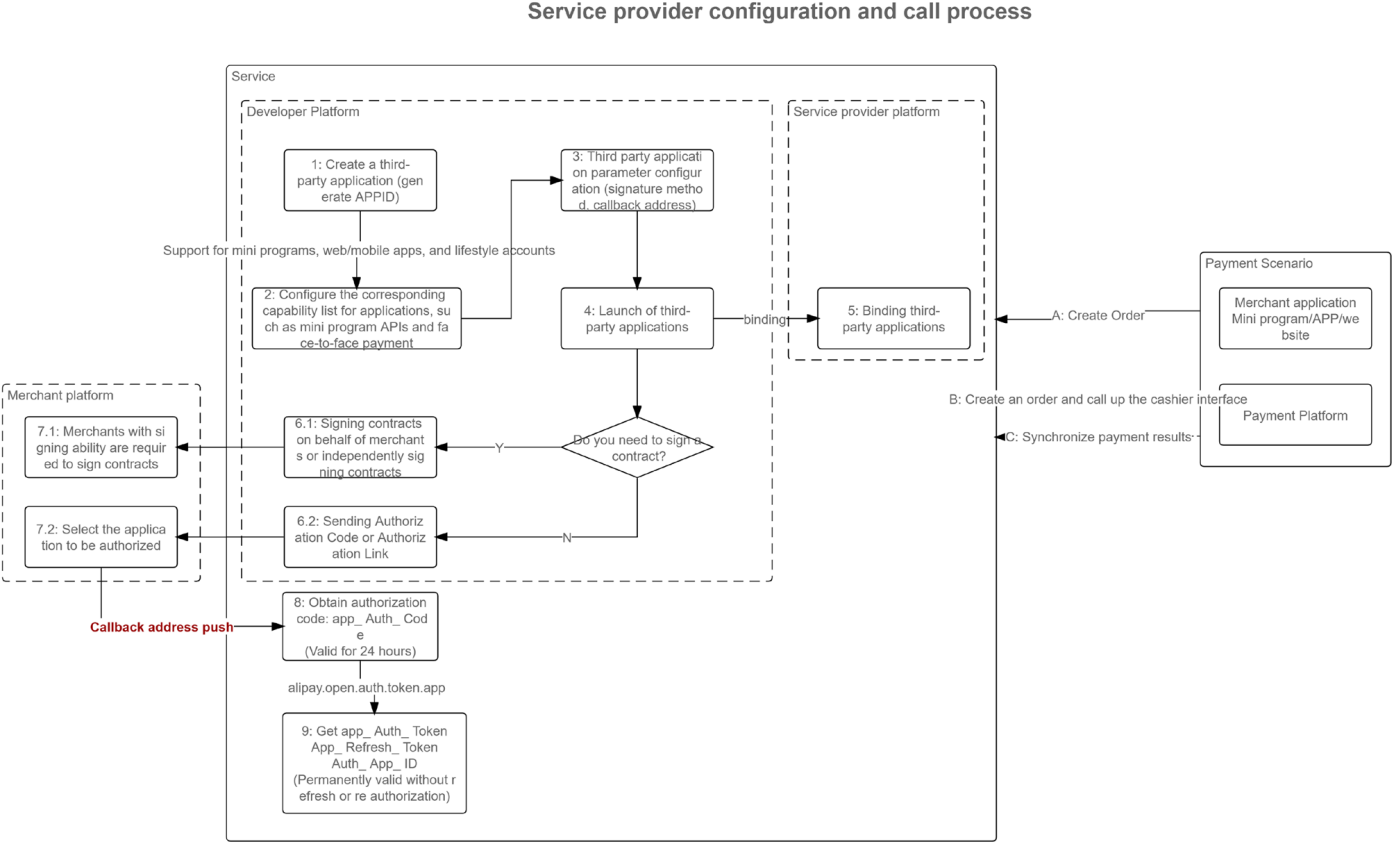
Full Link Application Architecture Parameter Transmission Details.

In Fig. 11, the logic of the application side full link sorting data interface is shown in the figure. On the left is a universal platform for multidimensional authorization and standardization of heterogeneous data. For the service layer, regular matching of third-party data sources is required, especially the logical verification of API interface message headers and information entropy. On the right is the API parameters for the binding interface of the service provider platform. For other third-party applications, payment and order placement are required, Facilitate the ecological construction of the original metaverse of the financial circle link cloud.

Before submitting, the business service sends events to the event service, which only records events and does not send them. The business service notifies the event service after submitting or rolling back, and the event service sends or deletes events. Don't worry about the business system crashing after submitting or rolling over, and unable to send confirmation events to the event service, because the event service will regularly retrieve all events that have not yet been sent and query the business system, and decide whether to send or delete the event based on the return of the business system.

Although external events can decouple the business system from the event system, they also bring additional workload: external event services incur two additional network communication costs compared to local event services (before submission and after submission/rollback), and the business system also needs to provide a separate query interface for the event system to determine the status of unsent events.

Precautions for reliable event notification mode:

There are two points to note about the reliable event pattern: 1 Correct sending of events; 2. Repeated consumption of events.By using asynchronous messaging services, it is possible to ensure the correct sending of events. However, it is possible for events to be sent repeatedly. Therefore, it is necessary for the consumer to ensure that the same event is not consumed repeatedly, in short, to ensure the idempotence of event consumption.

If the event itself is a state type event with idempotence, such as notification of order status (placed, paid, shipped, etc.), the order of the events needs to be determined. Generally, it is determined through a timestamp that after consuming a new message, when receiving an old message, it is directly discarded and not consumed. If a global timestamp cannot be provided, consideration should be given to using a globally unified serial number.

For events that do not have idempotence, they are generally action behavior events, such as a deduction of 100 or a deposit of 200. The event ID and event result should be persisted, and the event ID should be queried before consuming the event. If it has already been consumed, the execution result should be returned directly; If there is a new message, execute it and store the execution result.

**Figure 12:**
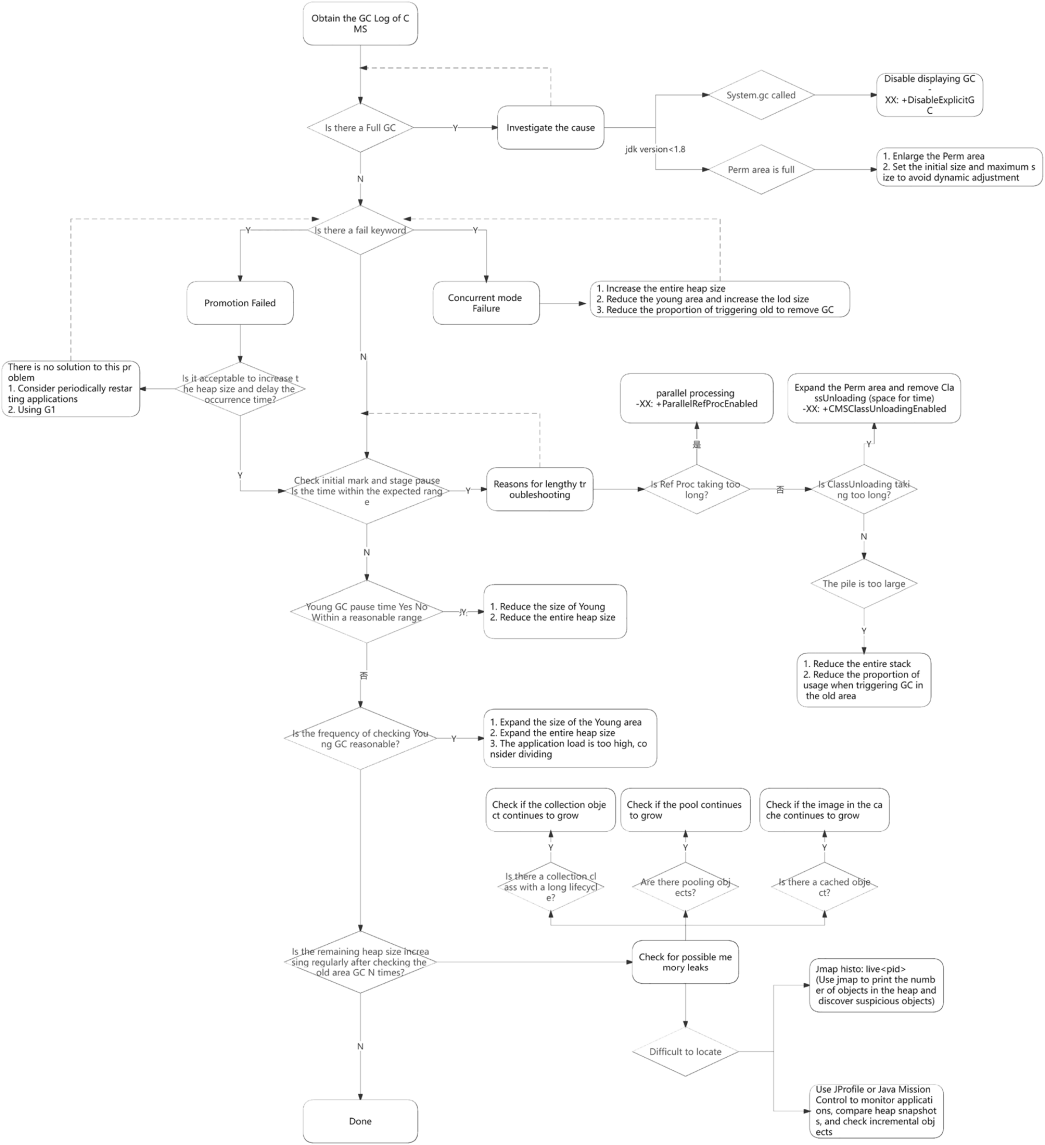
Metaverse Full Link Native Multi Thread High Concurrency Response Mechanism.

As show in Fig. 12, The details of the full link implementation are shown in the figure. Under the multi-dimensional mapping mechanism of each fitness multithreading, thread pooling operations are carried out, and the aggregation interface of local attribute categories is divided into different threshold ranges and priority settings. At the same time, multi-dimensional mapping is required for the protocol stack of multi thread program metaverse overflow, which facilitates optimization of high concurrency, high reliability, end-to-end latency, and load balancing in available scenarios.

### Monitor the status of GC

Use various JVM tools to view the current logs, analyze the current JVM parameter settings, and analyze the current heap memory snapshot and GC logs. Based on the actual memory partitioning and GC execution time of each region, decide whether to optimize.

For example, some phenomena before a system crash:The time for each garbage collection is getting longer and longer, from the previous 10 ms to around 50 ms, and the time for FullGC is also extended from the previous 0.5 s to 4-5 s.

The frequency of FullGC is increasing, with the most frequent occurrence occurring less than a minute apart,The memory of the older generation is getting larger and no memory is released after each FullGC,Afterwards, the system will be unable to respond to new requests and gradually reach the critical value of OutOfMemoryError. At this point, it is necessary to analyze the JVM memory snapshot dump.

### Generate a dump file for the heap

Generate the current Heap information through JMX's MBean, which is an hprof file of size 3G (the size of the entire heap). If JMX is not started, the file can be generated through Java's jmap command.

### Analyze the dump file

To open this 3G heap information file, it is obvious that typical Windows systems do not have such a large amount of memory and must use highly configured Linux and several tools to open the file:

- Visual VM
- IBM HeapAnalyzer
- Hprof tool included with JDK
- Mat (a specialized static memory analysis tool for Eclipse) is recommended for use

Note: The file is too large. It is recommended to use Eclipse's specialized static memory analysis tool Mat to open the analysis.

### Analyze the results to determine whether optimization is needed

If the parameters are set reasonably, there are no timeout logs in the system, the GC frequency is not high, and the GC time is not high, then there is no need to perform GC optimization. If the GC time exceeds 1–3 s or frequent GC, optimization is necessary.

Note: If the following indicators are met, GC is generally not required:

- The execution time of Minor GC is less than 50 ms;
- Minor GC execution is not frequent, approximately every 10 s;
- Full GC execution time is less than 1 s;
- Full GC execution frequency is not considered frequent, not less than once every 10 min.

### Adjust GC type and memory allocation

If the memory allocation is too large or too small, or if the GC collector used is relatively slow, priority should be given to adjusting these parameters, and one or several machines should be first selected for beta testing. Then, the performance of the optimized machine and the unoptimized machine should be compared, and targeted final choices should be made.

### Continuous analysis and adjustment

Through continuous experimentation and trial and error, analyze and find the most suitable parameters. If the most suitable parameters are found, apply these parameters to all servers.

**Figure 13:**
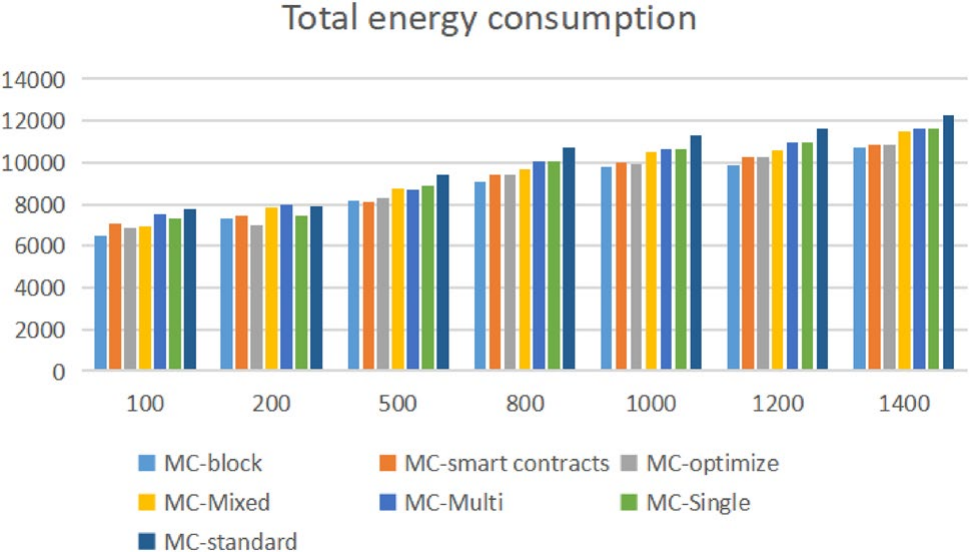
Total energy consumption.

As can be seen from Fig. 13, MC block represents the optimized Microservices security mechanism framework proposed in this paper. In the QPS range of 100, the energy consumption is lower than that of other typical algorithms, because in the low concurrency scenario, the gateway side and link tracking module have low space and time in the process of api encryption and decryption, and the input transcoding rate for the fitness function is low, so there is some performance improvement. However, with the increase of the workload, Especially in the interval [200500], the energy consumption of the optimization framework proposed in this article increases because the task volume in this interval needs to be further decomposed into itasks. For other algorithm frameworks, there is no need for transcoding point frame mapping mechanism, so the performance of other algorithms has advantages. However, as the level of data increases, the advantage of the multi-objective optimization framework for full link energy consumption performance proposed in this article is more obvious, with an average improvement of 5.38%

**Figure 14:**
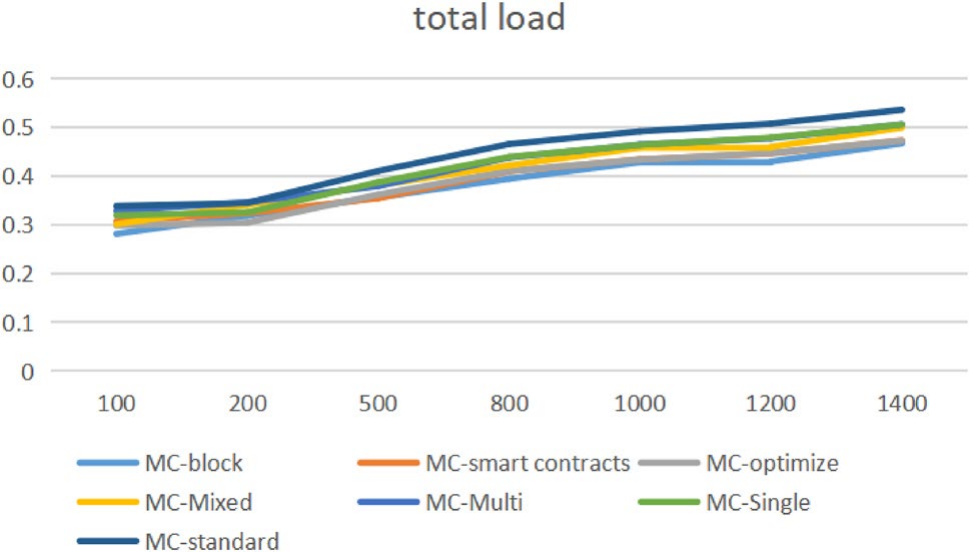
Full-link load balance.

From Fig. 14, it can be seen that within the [100150] interval, the proposed optimization algorithm has achieved a 3.8% improvement in local search and global convergence, reducing the load balance throughout the entire lifecycle. However, within the [200800] interval, the loss is relatively high due to the large number of task blocks in this segment and the diversification of structured semi structured data attributes, resulting in triggered multithreading issues in components such as fuse reduction in the registry, Load balancing requires re detecting the information entropy of each node based on the perceptual balancing algorithm. However, within the [8001400] interval, load balancing has gradually improved, with an improvement rate of 1.82%. In high concurrency scenarios, in addition to aggregation on the gateway side, the security filtering mechanisms for circuit breaker reduction, task arrangement, and cluster deployment stages are all placed in the multi-objective function optimization system, The number of invalid threads and coroutines is within the configured Thread pool range.

**Figure 15:**
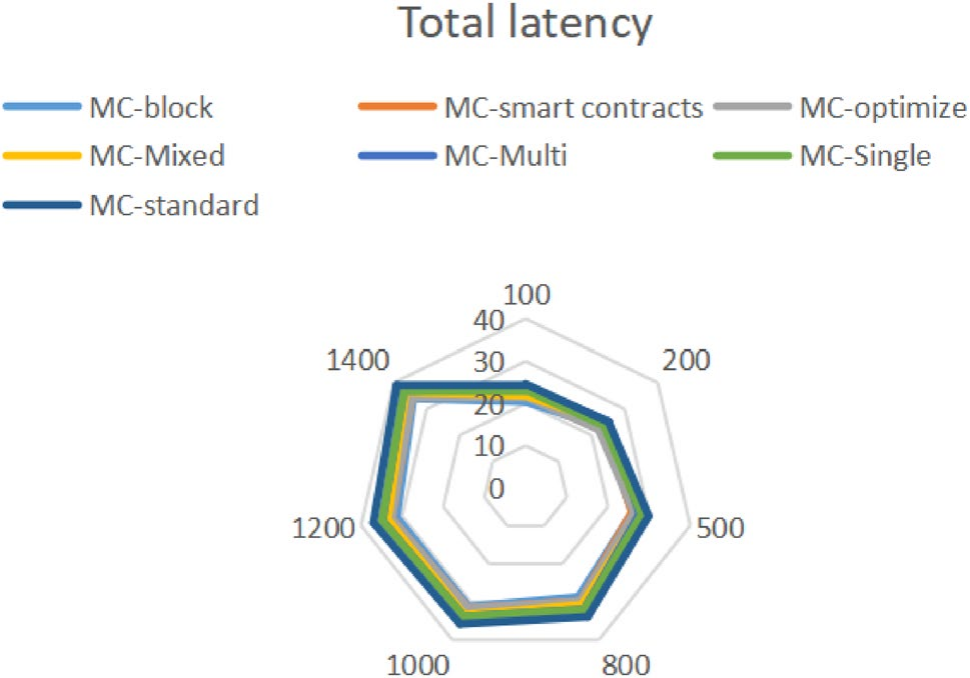
Full link delay.

From Fig. 15, it can be seen that the latency and MC block improvement of the entire link are all in the lower 7th level, because components with high latency dependency are safe detection mechanisms, and the reconstruction mechanism of the detection mechanism is defined in the custom definition of multi-objective functions. The quantization factor of multi-objective subfunctions is also the standard for quantifying the topology construction in this article, such as link tracking and traffic detection component modules. Therefore, there is also a filtering mechanism for global interception, The adaptation of the proxy mechanism is included in the encapsulated security proxy detection pool. Overall increase of 3.12%.

## Conclusions

We introduces the definition of a service maturity model proposed by mainstream frontline large corporations in the financial field, which evaluates the maturity of services using quantifiable indicators. Then, based on the model as a standard, the backend services were evaluated and the indicators with generally lower scores for each backend service were summarized. We have sorted out optimization plans for these low sub items. And an automated service quality detection mechanism has been established to ensure long-term stability of service maturity. Through availability optimization, the maturity model scores of multiple important services have been raised to over 8 points. Through an automated service quality detection mechanism, various indicators of maturity have been monitored for a long time. Once some indicators are found to be abnormal, they should be repaired and improved in a timely manner. In the future, continuous improvements and enhancements will be made in the following two areas: automation of maturity model scoring. Avoid subjective factors of manual grading while improving efficiency; The automated service quality detection mechanism adds more indicators. I hope to continuously improve the service availability of this study through optimization based on the service maturity model.

This paper proposes a multi-objective optimization Microservices framework that takes into account the security mechanism, Define the fitness function, define the upper and lower limits, and perform multi-dimensional constraints to filter for global and local optima. At the same time, reconstruct the circuit breaker current limiting mechanism, dynamically detect protocol parameters, and perceive the logical relationship of heartbeat status in real-time. Experimental results have shown that this innovative framework can solve scenarios such as high concurrency, high reliability, and high availability Performance bottleneck, business degradation, especially in financial and securities scenarios.

## Data Availability

The dataset generated and/or analyzed during the current research period is not publicly available, but can be obtained from corresponding authors upon reasonable request.
